# Psychometric evaluation of the Forensic Inpatient Observation Scale (FIOS) in youngsters with a judicial measure

**DOI:** 10.1186/1753-2000-5-30

**Published:** 2011-09-27

**Authors:** Chijs van Nieuwenhuizen, Ilja L Bongers

**Affiliations:** 1 Tilburg University, Tranzo - Scientific center for care and welfare, PO BOX 90153, 5000 LE Tilburg, the Netherlands; 2 GGzE Center for child & adolescent psychiatry, PO BOX 909, 5600 AX Eindhoven, the Netherlands

**Keywords:** juvenile delinquents, behavioral functioning, inpatients

## Abstract

**Background:**

In this article, the psychometric properties of the Forensic Inpatient Observation Scale (FIOS) were examined. This instrument was developed to observe behavioral functioning of forensic psychiatric patients. Up till now, it has only been used among adult forensic psychiatric patients and this is the first study in which the FIOS is used with youngsters.

**Methods:**

Data were gathered of 133 patients. The FIOS was routinely used to assess the psychiatric condition of youngsters at fixed intervals with a three-month time period between each measurement. Ward staff working in close contact with the patient conducted the assessments. Of these 133 patients, an YSR/ASR questionnaire was available for 96 of them and a TRF for 110 of the 133 patients. For the descriptive, reliability and validity analyses, SPSS version 16.0 was used. Factor analyses were performed by means of Mplus Version 5.2.

**Results:**

A series of confirmatory and exploratory factor analyses revealed a five-factor structure for the FIOS. The five-factor structure consisted of the following scales: self-care, social behavior, oppositional behavior, verbal skills and distress. The insight scale of the original factor structure could not be replicated in the youth sample. Cronbach's alpha's of the five scales ranged from .70 to .85. The self-care, verbal skills and oppositional behavior scales of the FIOS showed no relation with emotional and behavior problems reported by the patients themselves or their teachers. The distress scale of the FIOS did show a relation with the emotional problems reported by patients themselves and the social behavior scale with behavioral problems as reported by teachers.

**Conclusions:**

The internal consistency of the FIOS was sufficient and the factor structure in the present sample of youngsters was in general comparable to the original factor structure in an adult sample. Its value lies in the focus on behavioral functioning of youngsters with judicial measures. What remains to be seen is whether this instrument is sensitive enough to register all aspects of behavioral changes, whether the interrater reliability is sufficient, and whether it has predictive validity to relapse and recidivism.

## Background

Treatment evaluation within youth forensic mental health care is primarily focused on recidivism rates and symptom reduction [1,2]. For individual evaluation purposes, recidivism rates are not very enlightening because they are measured after treatment and are not related to therapy progress of the individual patient. Though symptom reduction is important for hospitalized youngsters, gaining insight into the improvement of their every day life skills and insight in their offence(s) is also important. Changes in these so-called dynamic variables are considered to prevent the individual from reoffending [3,4].

Group workers and nurses play an important role in facilitating change in dynamic variables. Van der Helm and colleagues [5] recently stated that 'support provided by group workers or staff, which builds on meaningful relationships and responsivity to the specific needs of each individual inmate, sets the groundwork for successful rehabilitation according to the 'Risks-Needs-Responsivity' principle.' So far, an instrument to measure behavioral functioning by group workers or nurses, however, is not available for youth forensic psychiatry. This article therefore focuses on the evaluation of an instrument to assess behavioral functioning: the Forensic Inpatient Observation Scale (FIOS; [6,7]). This instrument not only assesses psychiatric symptoms but also oppositional behavior and attitude towards offenses. Furthermore, the FIOS can be used to observe all forensic psychiatric patients and is not limited to a specific subgroup of offenses or diagnoses. Moreover, it refers to general behavior relevant to leading a life that is acceptable in society [7].

A major advantage of the FIOS is that it is a nurse-rated assessment tool of which not many exist in forensic psychiatry. The instruments that are available often focus on specific behavior such as aggression (e.g. Staff Observation Aggression Scale [8]; Observation Scale for Aggressive Behavior [9]) or are primarily developed for adult forensic psychiatric patients (e.g. Behavioral Status Index [10]). The use of a broader observation by ward staff working in close contact with patients is important since it offers insight into actual behavior as shown during the day. Often, behavior is measured using measures such as the Youth Self Report, the Adult Self Report and/or the Teacher Report Form [11,12], which might give conflicting results. Florsheim and colleagues [13], for instance, examined the role of working alliance in the treatment of delinquent boys focusing on clarifying the relation between therapeutic process and behavioral change. They used the Teacher Report Form (TRF) and the Youth Self Report (YSR) to describe the behavioral change. The TRF was filled in by ward personnel. The results from the TRF indicated changes on externalizing as well as on internalizing behavior that were related to long-term outcome. For boys, on the other hand, only changes on internalizing behavior were related to long-term outcome.

The aim of the present study was to evaluate the psychometric properties of the Forensic Inpatient Observation Scale (FIOS). More specifically, the study aimed to discover:

1. Whether the original factor structure of the FIOS, based on an adult sample, can be replicated in a sample of adolescents.

2. Whether the FIOS demonstrates adequate reliability and (convergent and divergent) validity in a sample of adolescents.

## Methods

### Patients

Data were gathered of patients admitted at Youth Forensic Psychiatric Hospital 'De Catamaran', the Netherlands. For a long time, the hospital has had a bed capacity of 28/29 beds. Currently, the bed capacity is 48-52 beds comprising six inpatient units of 8/9 beds each. The hospital offers both psychological and psychiatric assessments and treatment of boys between the age of 16 to 24 years who have been involved with the criminal justice system and/or pose a risk to themselves or to others through their behavior.

Observations were available for 133 patients, admitted to the hospital between September 2005 and December 2009. The mean age at admission was 17.3 years (range 14-22). Mean length of stay was 14 months (range = 1-48; sd = 10.8). Of the 133 patients, 70 were detained under civil law (53%) and 54 under criminal law (40%). Seven patients were admitted on a voluntary basis and for two patients the court order could not be traced (7%). Mean number of convictions was 1.6 (range 1-12). Of the total group, the largest group - that is 41 patients (31%) - had committed violent crimes. Other offenses were: arson (5%), sexual crimes (18%), homicide (2%), and (attempted) murder/manslaughter (3%). Only 31% (N = 41) had no criminal background. The psychiatric background of the total group, according to Axis-I classification of the DSM-IV, was: 13% schizophrenia and other psychotic disorders, 32% pervasive development disorder NOS or Asperger, 24% oppositional development disorder/conduct disorder, 5% ADHD and 18% other Axis-I disorders. A large proportion of the patients had a sub diagnosis of substance use/abuse of which cannabis (28%) and polydrug use (13%) were the largest groups.

### Instruments

#### Forensic Inpatient Observation Scale (FIOS)

The FIOS [6,7] was developed to assess the level of functioning of forensic psychiatric patients and is divided in six subscales: self-care (7 items), social behavior (6 items), oppositional behavior (10 items), insight offense/problems (4 items), verbal skills (3 items) and distress (5 items). The FIOS has been developed specifically for forensic psychiatric inpatients. One of the first steps in its development was the selection of treatment goals, based on treatment records, for adult forensic psychiatric patients and to combine these goals on a conceptual level with actual reported behavior of the patients in the daily treatment reports. Throughout the development process, clinicians were consulted for instance to evaluate items on their relevance for evaluating treatment progress and whether items comprised behavior observable to others. As a result, the FIOS does not focus on psychiatric symptoms per se, but on behavior that refers to general behavior which is considered relevant to leading a life without being a threat to self and/or others.

The original FIOS had appropriate internal consistency: Cronbach's alpha's ranged from .73 to .91 for the subscales. The convergent validity of the FIOS has been investigated in an earlier study by Timmerman et al. [7]. Results of this study showed that there was an association between the FIOS and several self-report measures and all relations were as hypothesized. The social behavior scale, for instance, correlated negatively with the anxiety and depression scale of the SCL-90 [14] and anxiety disposition of the State-Trait Anxiety Inventory (STAI [15]), whereas the distress scale correlated positively with the aforementioned scales of the SCL-90 and the STAI. The oppositional behavior scale correlated positively with the distrust and hostility scale of the SCL-90.

#### Youth Self Report (YSR) and Adult Self Report (ASR)

The YSR [11] is a questionnaire to be completed by youngsters of 11 to 18 years old, whereas the ASR [12] can be filled out by adults of 18 to 59 years. The YSR contains 120 items and the ASR 126 items. In both instruments, the items cover behavioral or emotional problems that occurred during the past six months. The response format for both questionnaires is: 0 = not true, 1 = somewhat or sometimes true, and 2 = very true or often true. The items of the YSR and ASR are summarized in two broad band scales pertaining to internalizing and externalizing problems and there is a total sum-score called the total problems scale. The reliability and validity of the ASR and YSR have been confirmed for the Dutch versions [16,17].

#### Teacher Report Form (TRF)

The TRF [11] comprises 120 items and has the same structure as the YSR and ASR. The Dutch version of the TRF also has good reliability and validity [18].

The YSR and ASR were used to obtain standardized reports of patients' problem behavior. The TRF was used to obtain standardized teacher reports of patients' problem behavior. In this study, the scores of the internalizing and externalizing problems scales of the YRS, ASR and TRF were used in the analyses. Using these scales, the divergent and convergent validity of the FIOS was tested.

### Procedure

In the first week of September 2005, the FIOS was introduced in our hospital. The FIOS is routinely used to assess the psychiatric condition of patients at fixed intervals with a three-month time period between each measurement. Ward staff working in close contact with the patient conducted the assessments. Staff members were informed both verbally and in writing and an instruction manual was developed. Three weeks before each assessment, a reminder was send by e-mail to inform the staff about the start of the observation period. Before the assessment, another reminder was sent. When the closing date approached, the response rate was checked and ward staff that had not yet responded, received a reminder by e-mail. All of the collected data were put in a datasheet. Using this procedure, the response rate up till now has been 100%.

Patients who received on-site schooling filled out the YSR or ASR in the same period that the staff filled out the FIOS and the teachers the TRF. The response rate for the YSR and ASR was approximately 81% (72-93%) and for the TRF 100%. Of the 133 patients with a FIOS-assessment, an YSR/ASR questionnaire was available for 96 of them and a TRF for 110 of the 133 patients. When the study was explained (verbally and in writing), written informed consent was obtained from each patient.

### Statistics

For the descriptive, reliability and validity analyses, SPSS version 16.0 was used. Factor analyses were performed by means of Mplus Version 5.2 [19]. Since the FIOS was originally developed for an adult sample, the factor structure for the adolescent sample was first investigated using a confirmatory factor analysis (CFA). The CFA was conducted in Mplus using the robust weighted least square (WLS) estimator (WLSMV) which is recommended for the analysis of skewed categorical data [20]. Each item was assumed to load on its own scale and scales were allowed to intercorrelate. Model fit was evaluated using the Bentler's comparative fit index (CFI; [21]), the Tucker-Lewis index (TLI; [22]) and the root-mean-square error of approximation (RMSEA; [23]). Patients that were admitted on a voluntary basis and from whom the court order could not be traced, were excluded from the analyses.

The exploratory factor analysis (EFA) of the FIOS was conducted in Mplus also using the WLSMV. Determination of the appropriate number of factors to be extracted, was based on the eigenvalues and interpretation of the factor structure. Based on the eigenvalues, we decided to systematically examine all possible factor solutions in EFA (i.e. from one to seven factors). The most promising model for EFA was subsequently examined by a confirmatory factor analysis (CFA). The factor solution of the five-factor EFA model was the most promising and was rerun in CFA and compared with the original factor structure of the FIOS that was based on an EFA in the adult sample [7]. Chi-square values were not reported for the CFA and EFA because they are difficult to interpret using WLSMV since the degrees of freedom are estimated. Consistent with Hu and Bentler [24], we adopted the criteria of RMSEA of .06 or below, or CFI and TLI greater than .90 as indicating a good fit with the proposed model.

Internal consistency was examined using Cronbach's alpha for the subscales in the two factor solutions. As guideline for evaluating Cronbach's alpha values as acceptable or not, Nunnally's [25] suggestion of .70 and above was used. Mean inter-item correlations were used as a measure of item homogeneity. Convergent and divergent validity were investigated using the YSR, ASR and TRF scores of the patients. Using the percentile scores of the normative sample of the non-referred children of the YSR, ASR and TRF on the internalizing and externalizing problems scales [11,12], the patients were classified in groups below the 25^th ^percentile (low group), between 25^th ^and 75^th ^percentile (medium group) and above 75^th ^percentile (high group).

The group differences on the FIOS were tested with one-way ANOVA with the FIOS scale scores of the five-factor structure as dependent variables and the groups on the YSR/ASR and TRF scales as independent variables.

## Results and discussion

### Confirmatory factor analysis

The goodness of fit indices for the original FIOS six-factor structure did not meet the required cut-off values. The CFI (.77) and TLI (.81) indicated that the model did not fit the data very well; also the RMSEA was above the cutoff (.159). Especially the insight scale showed a bad fit (see Table 1). Running the CFA without the items of the insight scale only marginally improved the fit (CFI = .82; TLI = .86; RMSEA = .147). Hence, exploratory factor analysis was justified.

**Table 1:** Confirmatory factor analyses of the original six-factor structure and the five-factor structure (EFA-version)

		*Original 6 factor structure*	*EFA 5 factor structure*
Self-care			
1	Wash himself	0.891	0.890
2	Brush teeth	0.944	0.942
3	Change clothes	0.903	0.903
4	Clean room	0.715	0.720
5	Cleaning duty	0.629	0.627
6	Day night rhythm	0.598	0.597
7	Dress himself	0.428	
Social behavior			
8	Present on ward	0.754	0.765
9	Group activities	0.823	0.828
10	Contact others	0.815	0.818
11	Initiate conversation	0.795	0.785
12	Talking experiences	0.688	0.661
13	Sociably present	0.727	0.738
Oppositional behavior			
14	Angry, irritated	0.793	0.775
15	Verbally aggressive	0.904	0.917
16	Utter threats	0.786	0.792
17	Pestering	0.411	
18	Lying	0.710	0.755
19	Sexual remarks	0.440	
20	Split the staff	0.708	0.723
21	Macho behavior	0.589	
22	Behaving overactive	0.590	
23	Recalcitrance	0.676	0.651
Insight			
24	Assertive criticism*	0.658	
25	Talk about offense	0.337	
26	Guilt toward victims	0.365	
27	Seriousness problems	0.732	
Verbal skills			
24	Assertive criticism*		0.649
28	Understand language	0.812	0.812
29	Talking audibly	0.688	0.688
30	Speaking Dutch	0.403	0.403
Distress			
31	Anxious, tense	0.854	0.982
32	Depressed, down	0.679	0.762
33	Stable mood	0.859	
34	Helpless, hopeless	0.613	0.612
35	Thoughts about suicide	0.515	0.617
			
CFI		0.81	0.90
TLI		0.85	0.93
RMSEA		0.14	0.114
% of explained variance		49.0	59.6

Note: * Item 24 loads in the original 6 factor structure on the Insight scale and in the EFA 5 factor structure on the Verbal skills scale.

### Exploratory factor analysis

The correlation matrix of the EFA showed that the first five factors had eigenvalues greater than 2 and factors 6, 7 and 8 had eigenvalues greater than 1 (see Table 2). On the basis of the interpretability and eigenvalues, the five-factor structure was seen as the most relevant model to examine in the CFA. There were no strong alternatives to the five-factor solution: the factor structures with three and four factors did not identify interpretable factors and the factors had large cross loadings. The six-factor structure created a factor with only two items. The EFA five-factor structure had a good enough fit to the data (CFI = .93; TLI = .95 and RMSEA = .085). The five-factor solution, which may be understood as a variant of the original six-factor structure, deviated from this model in three ways: (1) The insight scale could not be replicated, (2) item 24 loaded on the verbal skills scale instead of on the insight scale and (3) several items from the original scales had strong cross loadings. Especially the original items from the oppositional behavior scale had strong cross loadings with the social behavior scale (item 17 'pestering', item 19 'sexual remarks', item 21 'macho behavior' and item 22 'behaving overactive'). The original items from the distress scale had cross loadings with oppositional behavior (item 33 'stable mood'). Item 8 ('present on ward', original item social behavior) loaded on self-care, social behavior and distress.

**Table 2:** Eigenvalues of the exploratory factor analysis of the FIOS

*Number of factors*	*Eigenvalues*
1	7.729
2	5.963
3	3.820
4	2.903
5	2.377
6	1.734
7	1.240
8	1.135
9	0.957

### The EFA five-factor structure in CFA

The EFA five-factor structure run in CFA revealed a better fit to the data than the original six-factor structure (see Table 1). The items from the original insight scale and the item with the cross loadings were not incorporated in the CFA. The CFI (.90) and TLI (.93) indicate that the model fits the data well; both fit indices indicate that the fit of the model is significantly better than the null-model. The overall fit index RMSEA, however, indicates that the model describes the data only mediocre (RMSEA = .11).

### Internal consistency of the factor structure

The Cronbach's alpha of the original factor structure and the EFA five-factor structure were comparable for most scales, only the Cronbach's alpha of the verbal skills differed (see Table 3). The Cronbach's alpha for the original six-factor structure for verbal skills was .63 and for the EFA five-factor structure the Cronbach's alpha for verbal skills was .70. The item homogeneity coefficients were also comparable for the five- and six-factor solution.

**Table 3:** Internal consistency of the FIOS subscales

*Subscale *	*Original 6 Factor structure *			*EFA 5 Factor structure *		
	**Number of items**	**Cronbach's alpha**	**Item homogeneity**	**Number of items**	**Cronbach's alpha**	**Item homogeneity**

Self-care	7	0.84	0.41	6	0.85	0.50
Social behavior	6	0.85	0.48	6	0.85	0.48
Oppositional behavior	10	0.85	0.36	6	0.84	0.48
Insight	4	0.52	0.21			
Verbal skills	3	0.63	0.38	4	0.70	0.37
Distress	5	0.77	0.40	4	0.77	0.40

### Convergent and divergent validity

The patients were divided into three groups according to the norm tables of the YSR/ASR and TRF (see Table 4). In the general population, 25% scores in the low group of the YSR/ASR and TRF whereas in the present study, less than 10% of the patient scored in the low group: internalizing problems scale YSR/ASR N = 10 (10.4%); externalizing problems scale YSR/ASR N = 8 (8.3%); internalizing problems scale TRF N = 4 (3.6%); externalizing problems scale TRF N = 4 (3.6%). In the general population, 50% scores in the medium group (percentile score 25% to 75%) whereas in the present study between 29.1% and 49.0% scored in the medium group. Most patients scored in the high group of the YSR/ASR and TRF except for the internalizing problems scale of the YSR/ASR.

**Table 4:** Divergent and convergent validity of the FIOS

*FIOS*								
			**N of patients**	**Self-care**	**Social Behavior**	**Oppositional Behavior**	**Verbal Skills**	**Distress**

YSR/ASR	Internalizing	Low	10	23.0 (4.8)	19.3 (3.7)	13.5 (3.6)	13.6 (1.6)	7.1 (2.2)
		Medium	47	22.2 (4.9)	20.3 (4.1)	14.0 (4.1)	14.7 (2.0)	7.4 (2.0)
		High	39	22.3 (4.9)	20.0 (4.1)	14.7 (3.9)	14.7 (2.0)	8.8 (2.3)
		F-test		0.110	0.256	0.469	1.347	5.449**
	Externalizing	Low	8	23.6 (5.4)	18.4 (4.6)	12.3 (2.1)	14.9 (2.6)	7.0 (1.6)
		Medium	35	22.7 (5.2)	20.8 (4.3)	13.9 (3.8)	14.7 (2.0)	7.5 (1.8)
		High	53	21.8 (4.5)	19.8 (3.8)	14.7 (4.2)	14.5 (1.9)	8.4 (2.5)
		F-test		0.677	1.353	1.569	0.241	2.724
TRF	Internalizing	Low	4	21.3 (3.8)	19.0 (3.5)	13.5 (1.9)	15.5 (2.4)	7.8 (1.3)
		Medium	38	22.6 (4.9)	20.0 (4.4)	14.5 (4.3)	14.1 (2.1)	8.0 (2.1)
		High	68	22.2 (5.2)	19.9 (4.1)	14.3 (3.7)	14.5 (2.0)	7.9 (2.4)
		F-test		0.173	0.109	0.145	1.193	0.046
	Externalizing	Low	4	24.0 (2.2)	17.5 (1.3)	11.3 (1.7)	14.3 (2.5)	7.8 (1.9)
		Medium	32	22.5 (5.0)	18.4 (3.7)	13.8 (3.6)	14.9 (2.0)	7.8 (2.2)
		High	74	22.2 (5.1)	20.7 (4.2)	14.7 (4.0)	14.2 (2.0)	8.0 (2.3)
		F-test		0.263	4.290*	1.990	1.438	0.042

Note: * p < .05;** p < .01 For each FIOS scale, means and s.d. (in brackets) are presented for the three groups of the YSR/ASR and TRF.

In Table 4, the mean scores of the FIOS scales are depicted for the three groups (low, medium and high according to percentile scores of the YSR/ASR and TRF). No relations were found between self-care and verbal skills and the level of the internalizing and externalizing problems of the patients. Patients who had - according to the teacher - the most externalizing problems (high group) scored higher on the FIOS social behavior scale than patients in the medium group (F(2,109) = 4.29; p = 0.02). For oppositional behavior there was no relation between the internalizing and externalizing problems rated by the teacher (TRF) or patients (YRS/ASR) and ward personnel (FIOS). Patients in the high group of the internalizing problems scale of the YSR/ASR were rated higher on the distress scale of the FIOS compared to patients who scored in the medium group of the internalizing problems scale of the YSR/ASR (F(2,96) = 5.68; p = 0.01).

## Discussion

The results of this study show that the FIOS can be used in a population of youngsters and that it has, with some slight adjustments, good internal consistency and a stable factor structure. With the current version, 26 items, instead of the 35 items of the original version, seem sufficient enough to score the behavior of youngsters. The fact that the number of items is reduced, allows us to customize the instrument more for an adolescent population. For instance, by adding items dealing with family and peer influence and drug use.

This study also shows that, even after nearly four and a half years, the response rate is still one hundred percent. Of course, this result was not obtained without a hitch. As mentioned in the procedure, staff was informed verbally as well as in writing, a computerized instruction manual was available and much time and effort was spent on reminding. This means that, when using an observation-instrument, ample attention should be given to implementation aspects. Since behavior of youngsters towards staff members depends on the staff member as well as the situation, it is importance to use the same informant. This way, observer errors can be minimized as much as possible [7,26].

In order to test the validity of the modified FIOS, it was investigated whether the FIOS scales could differentiate between patients with different levels of emotional and behavioral problems. The FIOS was able to differentiate between patients who reported higher levels of emotional problems and lower levels of emotional problems. Whereas teachers were not able to classify the patients in distinctive groups based on their level of emotional problems. These results might imply that ward personnel is better equipped to observe emotional problems than teachers [27]. An interesting finding was that the level of behavioral problems of the patients at school only differentiated for social behavior and not for oppositional behavior on the ward. This can be explained by the fact that, on the ward, the social interaction between the peers plays an important role and thus is easier to observe. At school, on the contrary, the focus is more on the individual guidance of youngsters and less on group interaction [28].

This study is not without limitations. For example: the generalizability of the findings is limited to boys who were admitted in a youth forensic psychiatric hospital in the Netherlands. Hence, the study should be replicated in different samples (e.g., hospitalized youngsters without a judicial measure or hospitalized girls with and without a judicial measure) to assess the robustness of our findings and the applicability of the FIOS in other samples. Moreover, the sample size of our study is fairly small though the found factor structure seems to be a reliable measure of behavior according to the Cronbach's alpha, item homogeneity measures and the validity measures. A major limitation is that the interrater reliability was not assessed in this study. The reason for this is that we put a higher priority to having ward personnel in close contact with the patient to do the assessments. As a consequence, 73% of the patients were scored by one staff member only and therefore the interrater reliability could not be tested. This does not absolve us from the obligation to still conduct a study pertaining to the interrater reliability.

## Conclusion

In conclusion, the FIOS has shown to be an instrument with adequate internal consistency. Its value lies in the focus on behavioral functioning of youngsters with judicial measures. What remains to be seen is whether this instrument is sensitive enough to register all aspects of behavioral changes, whether the interrater reliability is sufficient, and whether it has predictive validity to relapse and recidivism.

## Competing interests

The authors declare that they have no competing interests.

## Authors' contributions

ChvN contributed to the conception and design of the study, helped with the interpretation of the data and prepared the manuscript; ILB performed the analyses and helped to draft the manuscript. All authors read and approved the final manuscript.
